# Utility of emergency call centre, dispatch and ambulance data for syndromic surveillance of infectious diseases: a scoping review

**DOI:** 10.1093/eurpub/ckz177

**Published:** 2019-10-12

**Authors:** Janneke W Duijster, Simone D A Doreleijers, Eva Pilot, Wim van der Hoek, Geert Jan Kommer, Marianne A B van der Sande, Thomas Krafft, Liselotte C H I van Asten

**Affiliations:** 1 Centre for Infectious Disease Control, National Institute for Public Health and the Environment (Rijksinstituut voor Volksgezondheid en Milieu, RIVM), Bilthoven, The Netherlands; 2 Department of Health, Ethics and Society, Care and Public Health Research Institute (CAPHRI), Faculty of Health, Medicine and Life Sciences, Maastricht University, Maastricht, The Netherlands; 3 Centre for Nutrition, Prevention and Health Services, National Institute for Public Health and the Environment (Rijksinstituut voor Volksgezondheid en Milieu, RIVM), Bilthoven, The Netherlands; 4 Julius Center for Health Sciences and Primary Care, University Medical Center Utrecht, Utrecht, The Netherlands; 5 Department of Public Health, Institute of Tropical Medicine, Antwerp, Belgium; 6 Institute of Geographic Sciences and Natural Resources Research, Chinese Academy of Sciences, Beijing, China; 7 Institute of Environment Education and Research, Bharati Vidyapeeth University, Pune, India

## Abstract

**Background:**

Syndromic surveillance can supplement conventional health surveillance by analyzing less-specific, near-real-time data for an indication of disease occurrence. Emergency medical call centre dispatch and ambulance data are examples of routinely and efficiently collected syndromic data that might assist in infectious disease surveillance. Scientific literature on the subject is scarce and an overview of results is lacking.

**Methods:**

A scoping review including (i) review of the peer-reviewed literature, (ii) review of grey literature and (iii) interviews with key informants.

**Results:**

Forty-four records were selected: 20 peer reviewed and 24 grey publications describing 44 studies and systems. Most publications focused on detecting respiratory illnesses or on outbreak detection at mass gatherings. Most used retrospective data; some described outcomes of temporary systems; only two described continuously active dispatch- and ambulance-based syndromic surveillance. Key informants interviewed valued dispatch- and ambulance-based syndromic surveillance as a potentially useful addition to infectious disease surveillance. Perceived benefits were its potential timeliness, standardization of data and clinical value of the data.

**Conclusions:**

Various dispatch- and ambulance-based syndromic surveillance systems for infectious diseases have been reported, although only roughly half are documented in peer-reviewed literature and most concerned retrospective research instead of continuously active surveillance systems. Dispatch- and ambulance-based syndromic data were mostly assessed in relation to respiratory illnesses; reported use for other infectious disease syndromes is limited. They are perceived by experts in the field of emergency surveillance to achieve time gains in detection of infectious disease outbreaks and to provide a useful addition to traditional surveillance efforts.

## Introduction

A relatively recent development in public health surveillance of infectious diseases is syndromic surveillance, which supplements conventional health surveillance systems by using less specific, near-real-time data from clinical and non-clinical sources to detect disease occurrence.^1^^,^^2^ Data sources include web searches, telephone helplines and over-the-counter medication sales.^1^ Syndromic surveillance allows almost immediate data collection, analysis and interpretation, which could lead to time gains as compared with traditional health surveillance.^1^^,^^3^^,^^4^ It offers cost effectiveness, flexibility and the potential to detect non-notifiable conditions as well as notifiable diseases overlooked by traditional surveillance systems.^3^^,^^5^ Usually syndromic surveillance does not use microbiological diagnoses, because they are not available soon enough (although advancing technology is decreasing the delay). This omission enhances system flexibility and rapidity but could diminish specificity, leading to excessive alerting.^6^ Another drawback is the lack of comparable syndrome definitions across time and place, which could hamper the data comparison and combination of multiple data sources needed for outbreak detection.^6^^,^^7^

Emergency medical service (EMS) data have been proposed for syndromic surveillance as it is based on predefined symptom definitions that are increasingly internationally standardized.^8^^,^^9^ Emergency department (ED) data have been widely used for syndromic surveillance. We focused on less commonly used EMS data, for which the value in infectious disease surveillance is less clear. The term EMS data here includes data collected during telephone calls made to emergency control centres that dispatch ambulances (e.g. 911 calls in the USA and 112 in Europe), hereafter called CC-dispatch data, and data collected by ambulance personnel at an emergency scene and/or during ambulance transport.^10^^,^^11^ There is also ambulance diversion data that results when EDs divert ambulances to other EDs due to overcrowding potentially caused by an infectious disease outbreak.^12^ Call-centre dispatch and ambulance are hereafter referred to as CCD&A. They provide valuable information on chief complaints and the spatiotemporal distribution of a subpopulation with a potentially severe disease or sequelae of infections who seek acute medical care. The data include cases that were not ultimately transferred to a hospital and thus not covered by ED data.^10^^,^^13^^,^^14^ Furthermore, each dispatch call is often triaged by a protocol-driven scheme and classified into standard call types, which could facilitate common symptom definitions among dispatch centrer.^13^^,^^15^ One example is the widely used Advanced Medical Priority Dispatch System (AMPDS), which ensures international standardization of calls with emphasis on the quality control of the dispatches [Reference 41 in Supplementary file S1]. As the primary goal of triaging emergency calls is allocation of resources, not making a diagnosis, the specificity of triage codes can be low.

Several countries have used CCD&A data for surveillance purposes, monitoring infectious diseases and non-infectious medical conditions, such as cardiac arrests or heat-related illness.^12^ Yet, to our knowledge, an overview of the literature on the added value of CCD&A-based syndromic surveillance for infectious disease detection is lacking. This study aims to provide such an overview and also reports on the utility of CCD&A data in infectious disease surveillance as perceived by key experts in the field.

## Methods

To evaluate CCD&A systems and their utility for infectious disease surveillance, a scoping review based on the framework of Arksey and O’Mally was conducted.^16^ This type of review is particularly useful in research areas where a comprehensive overview of the available literature is lacking. According to the framework, a scoping review differs from a systematic review in having a broader research question.^17^ Besides, there is room for redefinition of search terms during the search process. Optionally, a scoping review can include a grey literature search and information from interviews with key informants. Further, unlike systematic reviews, such studies are usually not subjected to formal quality assessment, allowing inclusion of a wider range of study designs.^16^ We conducted a literature review (peer-reviewed and grey publications) and interviews with experts in the field.

### Identifying relevant articles

The literature review was initiated by multiple explorative searches in Embase, PubMed and Google (Scholar) to define the search terms. Subsequently, PubMed was searched for peer-reviewed publications. The search terms were divided into three groups relating to emergency care (i.e. ambulance, dispatch), infectious diseases (i.e. infection, outbreak) and public health surveillance [i.e. (syndromic) surveillance, early warning, monitoring] (Table A in Supplementary file S2). Within each group, search terms were linked with Boolean operator ‘OR’, and the groups were combined using Boolean operator ‘AND’. All English language articles published from 1990 to June 2018 with titles or abstracts describing CCD&A data in the context of infectious disease surveillance were considered potentially relevant.

The initial internet search revealed some potentially useful records in the grey literature, i.e. not scientific journals. A targeted internet search for those was then performed using the predefined search terms (Table A in Supplementary file S2). As it produced many irrelevant hits, we limited the search to reports from organizations in the field of syndromic surveillance known to us that provided additional links: FirstWatch (a commercial surveillance company), the International Society for Disease Surveillance (ISDS), Triple-S (inventory of European syndromic surveillance initiatives) and SIDARTHa (European project exploring emergency data-based syndromic surveillance). Similar to the scientific review, the focus was on English language articles from 1990 to June 2018 describing CCD&A data in the context of infectious disease surveillance. A broad range of publication types were explored, including news items, webinars, conference abstracts and project reports. The results from this search are hereafter referred to as ‘grey publications’.

Supplementary file S2 details the search strategy, selection of articles, and charting and collation of information for both the peer-reviewed and the grey literature.

### Collating, summarizing and reporting results

The summary of the selected literature was structured based on several elements of the Framework for Evaluating Public Health Surveillance Systems for Early Detection of Outbreaks: study characteristics (e.g. study purpose), timeliness and the validity of the system.^18^

### Key informant interviews

Interviewees were purposively selected based on their professional experience in EMS syndromic surveillance as reflected by their publications or employment in the field. Mostly identified through the literature search or through contacts in the Dutch network of ambulance care, 25 potential participants were contacted via e-mail. Contacts included researchers, EMS-healthcare workers or CCD&A-based syndromic surveillance personnel and they received the interview questions with a letter explaining research aims, interview procedures and privacy matters.

One researcher (S.D.) conducted all interviews with the 11 consenting participants: 5 by Skype, 2 by telephone, 1 face-to-face and 3 by written communication. Conversation time averaged 37 min (range 20–58 min). All oral interviews were recorded and transcribed verbatim. As telephone interviews are prone to technical difficulties and lack non-verbal communication, transcripts were sent to each participant for approval. Data analysis was performed by one researcher (S.D.) using Atlas.ti version 7.5.6 and an inductive coding strategy (i.e. using themes that emerged from the interviews instead of pre-conceived themes).

## Results

### Literature review

**Figure 1:**
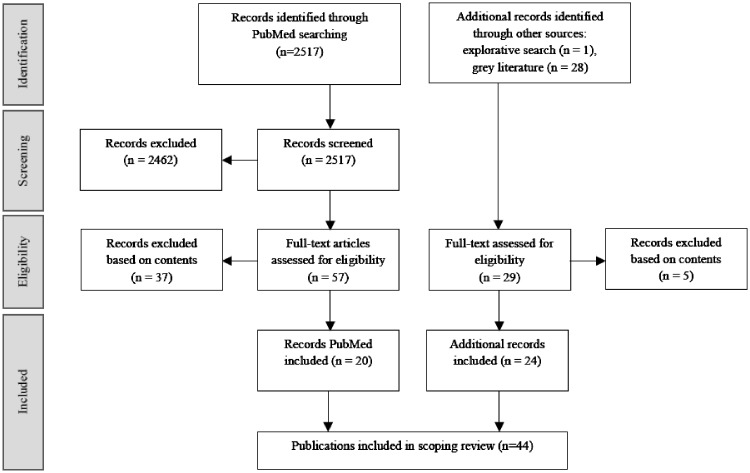
PRISMA flow diagram of publication selection process

The search strategy yielded 2546 records for peer-reviewed and grey literature combined (figure 1). Full-text assessment was performed on 86 records, of which 44 were included in the final scoping review.

#### Study characteristics

**Figure 2:**
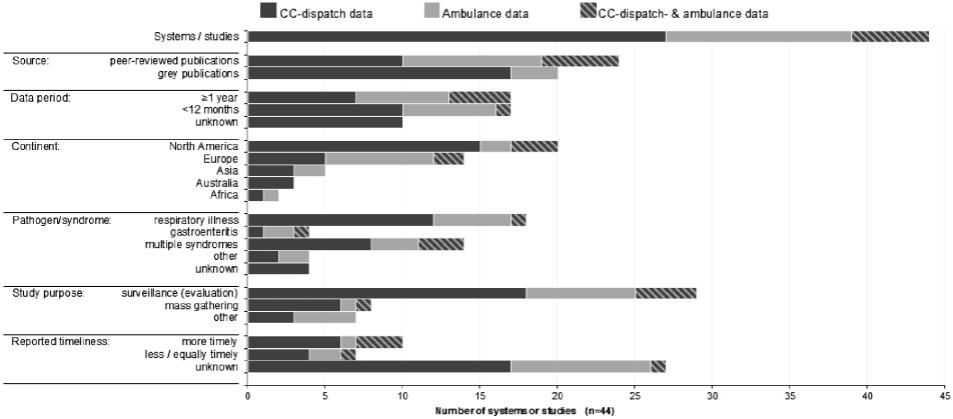
Summary of study characteristics and main outcomes of the systems as described in peer-reviewed publications and grey publications (*n* = 44)

*Peer-reviewed publications*. Published between 2003 and 2017, the 20 peer-reviewed publications described 24 systems or studies and were conducted in countries classified as ‘high-income’ by The World Bank: Austria, Australia, Belgium, Canada, Denmark, Germany, Italy, Japan, Poland, the UK and the USA (table 1 and figure 2). Of these systems/studies, 10 assessed CCD&A-based syndromic surveillance, mostly related to respiratory infections;^10^^,^^15^^,^^19–22^^,^^24^ [References 46, 50, 51 in Supplementary file S1]; 5 studies used CCD&A-based surveillance for situational awareness at high-profile events;^23^ [References 42, 47–49 in Supplementary file S1], and 5 used CCD&A data for other purposes including estimating the burden of influenza on EMS-workload;^12^^,^^25^ [References 43–45 in Supplementary file S1]. Of the 24 systems/studies, 14 were retrospective, 9 were prospective and 1 combined both (table 1 and Supplementary table S1). This last system, described by Bork et al., has become an active system in Denmark named BioAlarm.^19^ Studies were executed between 1995 and 2016 (Supplementary table S1) over periods ranging from under 1 month (n = 4) or several months (n = 10) to multiple years (n = 10). Of the 24 systems, 10 used CC-dispatch data, 5 used ambulance data and 9 combined both data types (figure 2). The only 4 studies that specified their dispatch triage system were all using AMPDS. For ambulance data, 2 used the 9th or 10th revision of the International Classification of Diseases (ICD-9, ICD-10) and 8 did not report the coding system (Supplementary table S1). Eleven studies provided information about the detection algorithm and/or the underlying suite of methods, which included CUSUM (n = 4), Early Aberration Reporting System (EARS) (n = 2), FirstWatch commercial software (n = 1) or a custom-developed (regression) model (n = 4).

**Table 1:** Overview of relevant peer-reviewed publications and their aims

First author, year	Location	Aim	Study design
Ayala, 2016 [Reference 42 in Supplementary file S1]	USA (Arizona)	Describe the enhanced epidemiologic surveillance efforts in place during Super Bowl XLIX and related events	Prospective
Bork, 2005 [19]	Denmark	Testing an active dispatch-based disease detection system aiming to reduce outbreak detection time (BioAlarm)	Retrospective (8 days prospective)
Brunetti, 2015 [Reference 43 in Supplementary file S1]	Italy (Apulia)	Reporting the impact of acute cardiovascular disease on EMS—workforce during the 2014–2015 influenza season	Retrospective
Brzezińska-Pawłowska, 2016 [Reference 44 in Supplementary file S1]	Poland (Lodz)	Assessing the association of severe exacerbations of asthma and COPD requiring an ambulance with meteorological parameters and influenza outbreaks	Retrospective
Coory, 2009 [20]	Australia (Melbourne)	Assessing if dispatch data could provide an alternative to sentinel ILI surveillance	Retrospective
Cretikos, 2009 [Reference 45 in Supplementary file S1]	Australia (New South Wales)	Presenting an overview of the progression of the 2009 H1N1 pandemic	Prospective
Fishbein, 2010 [Reference 46 in Supplementary file S1]	USA (El Paso)	Evaluating the use of EMS dispatch and response logs to detect infected travellers	Prospective
Foldy, 2004 [Reference 47 in Supplementary file S1]	USA (Milwaukee)	Provision of situational awareness (including infectious outbreak detection) on several high profile entertainment events and improvement of emergency communications among health providers	Prospective
Franke, 2006 [Reference 48 in Supplementary file S1]	France (Briançon)	Provision of situational awareness (including infectious outbreak detection) and guidance of interventions during 2006 Winter Olympic Games	Prospective
Greenko, 2003 [15]	USA (New York City)	Examining biases in ambulance dispatch data, as well as case sensitivity and predicting value positive of ILI call types	Retrospective
Haas, 2011 [21]	USA (Cincinnati)	Testing automated surveillance based on dispatch data for water contaminations	Prospective
Mostashari, 2003 [22]	USA (New York City)	Evaluating dispatch data as a data source for respiratory illness surveillance	Retrospective
Ohkusa, 2011 [Reference 49 in Supplementary file S1]	Japan (Toyako)	Provide a description of the syndromic surveillance systems used during the G8 Hokkaido Toyako summit meeting 2008	Prospective
Polkinghorne, 2011 [Reference 50 in Supplementary file S1]	Australia (Sydney)	Assessing the association between febrile convulsions in young children and seasonal influenza	Retrospective
Rosenkötter, 2013 [Reference 51 in Supplementary file S1]	Austria (Kufstein district), Belgium (Leuven)	Evaluating the performance of syndromic influenza surveillance based on dispatch and ambulance data	Retrospective
Schull, 2004 [12]	Canada (Toronto)	Exploring the relationship between ED crowding and influenza outbreaks (based on ambulance diversion hours)	Retrospective
Shimantani, 2015 [23]	Japan (Tokyo metropolitan area)	Provision of situational awareness (including infectious outbreak detection) at the 2013 Sports Festival in Tokyo	Prospective
Todkill, 2017 [24]	England (West Midlands region)	Determine the feasibility and utility of using ambulance data as part of routine syndromic surveillance activities	Prospective
Tsubokura, 2010 [25]	Japan (Kobe)	Impacts of H1N1 influenza pandemic on EMS	Retrospective
Ziemann, 2014 [10]	Austria (Tirol), Belgium, Germany (Göppingen)	Developing and testing a concept for syndromic surveillance, based on dispatch- and ambulance data	Retrospective

EMS, emergency medical services; COPD, chronic obstructive pulmonary disease; ILI, influenza-like illness.

*Grey literature publications*. The grey literature search within SIDARTHa, Triple-S, ISDS and FirstWatch publications resulted in 24 records describing an additional 20 CCD&A-based syndromic surveillance systems or studies (figure 1 and table 2). The SIDARTHa search found no system descriptions beyond what was already found in peer-reviewed literature. The remaining publications described currently active syndromic surveillance systems (*n* = 13) and research on the performance and utility of CCD&A data (*n* = 5), mostly addressing respiratory infections or gastrointestinal illnesses (figure 2 and table 2). Triple-S publications described two planned syndromic surveillance systems (Belgium, Hungary) and one planned feasibility study in the border region of Belgium, the Netherlands and Germany (Euroregio Maas-Rhine) [References 71, 72 in Supplementary file S1]. Surveillance systems were operating mainly in high-income countries but also Uganda, Haiti and India (ISDS). The aims of CCD&A-based syndromic surveillance systems (active and planned) included infectious disease detection and generation of situational awareness at mass gatherings, including infectious disease outbreak detection [References 52–73 in Supplementary file S1] (table 2 and figure 2). All FirstWatch syndromic surveillance systems were prospective, with one 2-month drop-in system serving the Republican and Democratic National Conventions (USA) [Reference 61 in Supplementary file S1]. Publications from ISDS conferences included two prospective systems and three retrospective studies ranging from several months to more than 2 years. The 2-year data in the study of Jena et al. (2010) from India was used in the model phase of a sequel study, published in 2012 [References 65, 66 in Supplementary file S1]. The four studies found via Triple-S consortium were based on data periods less than 1 year. CC-dispatch data was the most used data type among all grey literature publications (*n* = 17) (Supplementary tables S2–S4). Ambulance data were used in the Uganda and Haiti systems and the planned system in Belgium. No system used a combination of both CC-dispatch and ambulance data. Details such as data coding system, detection method or reference data were lacking in many publications. The use of AMPDS combined with electronic Patient Care Records (ePCRs) was reported for three CC-dispatch-based systems, all in the USA (FirstWatch *n* = 2; ISDS *n* = 1) [References 55, 63, 68, 69 in Supplementary file S1]. Use of ICD-9 and ICD-10 was reported in two Triple-S publications about Belgium and Hungary [References 72, 73 in Supplementary file S1]. Detection methods were provided for five systems, being CUSUM (*n* = 4) and EARS/SaTScan (*n* = 1) combined with regression analysis or spatial analysis [References 54, 64, 66, 72, 73 in Supplementary file S1] (Supplementary tables S2–S4).

**Table 2:** Overview of relevant grey publications and their aims

Category	First author/organization, year	Publication type	Country	Aim	Active system/study
FirstWatch	*USA Today*, 2003 [Reference 52 in Supplementary file S1]	Newspaper article	USA	Detecting influenza outbreaks	Active
	Barishansky, 2005 [Reference 53 in Supplementary file S1]	Article in emergency medical services	USA	Detecting influenza outbreaks	Active
	FirstWatch, 2007 [Reference 54 in Supplementary file S1]	FirstWatch brochure	USA	Detecting influenza and gastrointestinal outbreaks	Active
	Scott, 2008 [Reference 55 in Supplementary file S1]	Article in *Emergency Number Professional Magazine*	USA	Detecting influenza outbreaks	Active
	Simon, 2009 [Reference 56 in Supplementary file S1]	Article in best practices in emergency medicine	Canada	Identifying pandemic influenza cases	Active
	FirstWatch, 2016a [Reference 57 in Supplementary file S1]	News items	USA	Detecting influenza outbreaks and bioterrorism attacks	Active
	FirstWatch, 2016b [Reference 58 in Supplementary file S1]	FirstWatch case study	USA	Detecting influenza outbreaks	Active
	Stout, 2011 [Reference 59 in Supplementary file S1]	Interview	USA	Provision of situational awareness (including infectious outbreak detection) at conference attended by Barack Obama	Active
	Eyewitness News, 2013; Goodwin, 2013a; FirstWatch, 2016c [Reference 60–62 in Supplementary file S1]	News item, article in *Journal of Emergency Medical Services*, FirstWatch case study	USA	Provision of situational awareness (including infectious outbreak detection) at 2013 Mardi Grass and Super Bowl	Active
	Goodwin, 2013b [Reference 63 in Supplementary file S1]	Article in *The Journal*	USA	Provision of situational awareness (including infectious outbreak detection) at the 2012 Republican and Democratic national conventions	Active
ISDS conference	Cairns, 2011 [Reference 64 in Supplementary file S1]	Conference abstract	USA	Developing a statewide CCD&A-based bio surveillance system	Study
	Jena, 2010 [Reference 65 in Supplementary file S1]	Article in *Indian Emergency Journal*	India	Analyzing the demand pattern of EMS services for fever in three selected districts of Andhra Pradesh, India	Study
	Jena, 2012 [Reference 66 in Supplementary file S1]	Webinar	India	Developing and testing a syndromic surveillance system based on dispatch data	Study
	Taylor-McCabe, 2013 [Reference 67 in Supplementary file S1]	Webinar	Uganda	Monitoring Ebola	Active
	Taylor-McCabe, 2013 [Reference 67 in Supplementary file S1]	Webinar	Haiti	Monitoring cholera	Active
	Stout, 2015; Garza, 2015 [References 68, 69 in Supplementary file S1]	Conference abstract, webinar	USA	Identifying potential Ebola cases	Active
Triple-S	D’Ortenzio, 2009; Vilain, 2011; [References 70, 71 in Supplementary file S1]	Article, conference abstract	Réunion Island	Demonstrating the complementarity of GP, dispatch and emergency department data in influenza surveillance	Study
	Ziemann, 2013 [Reference 72 in Supplementary file S1]	Triple-S report	Border region of Belgium, the Netherlands and Germany	Testing feasibility of CCD&A data sources and establishing proof of concept for syndromic surveillance	Study
	Conti, 2012 [Reference 73 in Supplementary file S1]	Triple-S report	Belgium	General public health surveillance	n/a
	Ziemann, 2013; Conti, 2012; [References 72, 73 in Supplementary file S1]	Triple-S reports	Hungary	Unspecified	n/a

ISDS, International Society for Disease Surveillance; CCD&A, call-centre dispatch and ambulance; EMS, emergency medical services; GP, general practitioner, i.e. primary care; n/a, not applicable.

#### Timeliness of outbreak detection

*Peer-reviewed publications*. The timeliness of the used data source in terms of outbreak detection or following trends during epidemics was addressed for 14 systems or studies (figure 2). Timeliness was quantified against reference data in only six, all investigating the use of CC-dispatch data (*n* = 5) or ambulance data (*n* = 1) and all in the context of respiratory infections;^20^^,^^22^^,^^24^^,^^27^ [References 45, 50, 51 in Supplementary file S1]. Syndromic respiratory alerts were often reported to be ahead of those based on hospital data (New South Wales and Sydney, Australia: *t* = −1 week) [References 45, 50 in Supplementary file S1], laboratory data (New South Wales: *t* = −1 week; New York City, USA: *t* = –2–3 weeks);^22^ [Reference 45 in Supplementary file S1], and general practitioner/primary care) data (Belgium: *t* = −1 week; Melbourne, Australia: *t* ≈ −8, 5 weeks);^20^^,^^27^ [References 51 in Supplementary file S1]. However, the 2-month time gain in Melbourne was questionable, as peaks occurred outside the influenza season and no reference data were available during the non-influenza period.^20^ In one study, influenza-like illness (ILI) aberration was detected in ambulance data slightly later than in reference data (Belgium, *t* = +2 days);^27^ [Reference 51 in Supplementary file S1], and several known outbreaks (mainly gastrointestinal) were undetected in CCD&A data in two studies.^10^

*Grey* *literature publications.* Information about reference data was provided for only three systems reported in grey literature; hence timeliness could not be assessed for most publications, including all FirstWatch publications (figure 2). Regarding publications from ISDS conferences, timeliness was quantified in one Indian study where CC-dispatch data, uploaded every 2–3 h, detected a dengue outbreak sooner (*t* = −15 days) than the reference data (newspaper articles) (Supplementary table S3) [Reference 66 in Supplementary file S1]. A publication describing an American study claimed large time gains in detecting a gastrointestinal event (*t* = −56 to −7 days), but no reference data were specified [Reference 64 in Supplementary file S1]. In Triple-S publications, timeliness was assessed only for the study on Réunion Island, where no time gain in influenza detection through CC-dispatch data were observed compared to sentinel primary care data (Supplementary table S4) [Reference 69 in Supplementary file S1].

#### Validity aspects and aberration detection

*Peer-reviewed publications*. Validity is the ability of the surveillance system to detect an actual outbreak, and its assessment requires an outbreak definition, including case definitions and detection algorithm results. Of 12 studies aimed at outbreak or aberration detection, 8 provided information about the detection algorithm (Supplementary table S1);^10^^,^^19–22^^,^^24^^,^^26^ [References 48, 49, 51 in Supplementary file S1]. Other relevant information for assessment of validity includes data completeness, outbreak and data characteristics (e.g. seasonal variation, responsible infectious agent), and response tools. In most studies, this information was incomplete. Statistical assessment of validity includes the calculation of sensitivity, specificity and positive predictive value (PPV); only two studies provided one or more of these;^15^^,^^27^ [Reference 51 in Supplementary file S1]. In the study of Greenko et al. in New York, the sensitivity of selected call types potentially related to ILI was 58%, with a PPV of 22%.^15^ The study of Rosenkötter et al. was focused on influenza syndromic surveillance in Europe and resulted in calculated sensitivities of 71.4% and 60.0% and specificities of 76.3% and 100% for CC-dispatch data and ambulance data, respectively.^27^ Other studies addressed the perceived sensitivity or utility but did not quantify these indicators. Generally, surveillance based on CCD&A data was reported by the respective authors as being a useful addition to existing surveillance systems in terms of timely detection of signals of suspected outbreaks of infectious diseases;^10^^,^^15^^,^^19–24^^,^^26^ [References 46, 47, 49–51 in Supplementary file S1]. However, aberration detection based on CCD&A data appeared less adequate for *continuing* outbreaks with low case numbers, especially given a large geographical spread.^10^^,^^19^^,^^22^

##### Grey literature publications.

Relevant information for assessment of validity was scarcely described in the grey literature. However, one ISDS webinar about an Indian study testing CCD&A-based syndromic surveillance to detect dengue reported the manner of baseline estimation, the nature of the dengue outbreak and factors influencing data quality, such as the extent of missing data (Supplementary table S3) [Reference 66 in Supplementary file S1]. Two publications concluded that CCD&A-based surveillance could strengthen the existing surveillance in terms of early detection of outbreaks or illnesses [References 64, 65 in Supplementary file S1]; the remaining publications drew no conclusion about the utility of the data used.

### Interviews

#### Characteristics of interviewees

Of the 25 invited participants, 11 agreed to an interview (response rate = 44%); 12 did not respond and one responded but ceased communication. Two interviewees each invited a colleague to join the conversation, so the 11 interviews involved 13 persons, most from Europe and the USA. Researchers, EMS-healthcare workers and CCD&A-based surveillance personnel were equally represented. All respondents had experience with CCD&A-based syndromic surveillance through research or an active system.

#### Usefulness

Most respondents (*n* = 11) considered CCD&A-based syndromic surveillance useful (Supplementary table S5). They focused mainly on its potential to detect aberrations in (emerging) infectious disease occurrences, *provided that* outbreaks were large and severe enough (*n* = 9). Its complementarity to primary care and ED data was seen as an important asset (*n* = 3), as it covers previously unmonitored populations (e.g. people seeking acute care who are not ultimately hospitalized). Few doubted the utility of CCD&A data (*n* = 3), but some (*n* = 2) compared it (unfavourably) with ED data, believing the latter to be more timely and more specific.

#### Attitude toward CC-dispatch data and ambulance data

The potential timeliness of CCD&A-based syndromic surveillance was perceived by most (*n* = 9) as its greatest benefit, due to the real-time availability of data collected early in the disease course (Supplementary table S6). Some participants (*n* = 2) felt that timeliness would be crucial for outbreak control. The systematic and electronic collection of CCD&A data was seen as cost efficient and convenient (*n* = 4). Some interviewees stated that only severe cases would appear within EMS, and that data coverage would thus be low (*n* = 3).

Advantages specific to CC-dispatch data mainly focused on its standardization, which was thought to enhance data quality and the user-friendliness of CCD&A-based syndromic surveillance (*n* = 3). An important disadvantage of CC-dispatch data was its perceived dependency on laypersons’ perceptions of emergencies (*n* = 5) that could lead to inaccurate data and public unrest. For example, two US respondents explained that people’s fear of Ebola, along with the cautiousness of EMS, led to an overestimated number of possible Ebola cases.

Ambulance data were considered more reliable than CC-dispatch data (*n* = 4) due to on-scene ambulance personnel and diagnostic equipment. Professional, as opposed to laypersons assessment of emergency telephone calls, increased confidence in this data type. Major pitfalls mentioned were non-standardization of ambulance data (*n* = 5) and variation in reporting quality of data by ambulance personnel (*n* = 3).

#### Data integration

Data integration in the context of EMS involves the standardization of definitions and data structures within and across data sources (e.g. CC-dispatch data and ED data), improving data quality and hence supporting outbreak detection. The lack of data integration of both CC-dispatch data and ambulance data was judged problematic (*n* = 5). Moreover, ambulance data across providers or systems were not always unified due to a lack of standardization that can hamper comprehensive assessment of outbreaks.

#### Alternative uses

All participants suggested alternative uses for CCD&A data (Supplementary table S7), especially the use of both data types in situational awareness (*n* = 5) and in optimizing EMS logistics (*n* = 6). Moreover, CCD&A data could aid in resource allocation, which would be of particular importance for large outbreaks (*n* = 5).

#### Improvements

The main themes included improving data integration (*n* = 5) and improving EMS facilities (*n* = 3) (Supplementary table S7). Upgraded diagnostic tools on ambulances were considered desirable to obtain more accurate diagnoses and thereby enhance data specificity. Two participants doubted that further improvements could be easily realized, as syndromic surveillance was not the main purpose of CCD&A data collection.

## Discussion

Our scoping review found that several CCD&A-based syndromic surveillance systems or studies have been in place/performed for infectious disease surveillance, but that approximately half of them are not documented in peer-reviewed journals. There were 20 peer-reviewed publications describing 24 systems/studies and 24 grey publications describing 20 additional systems/studies. Most studies were retrospective, suggesting that prospective continuous use of CCD&A data in syndromic surveillance is not yet well established or not reported. Few active systems were described in peer-reviewed or grey literature. All studies comparing trends in CCD&A data with trends in pathogen circulation were ecological studies; no validation was provided as to what extent observations in CCD&A data can actually be linked to causative infectious agents.

The main aims of the surveillance systems/studies were similar across the 44 publications. However, grey publications provided less detail about the methods and utility of CCD&A-based syndromic surveillance. A few provided general information about using emergency data for infectious disease surveillance but did not meet our inclusion criteria.^28–30^

Characteristics of the reported CCD&A-based syndromic surveillance studies/systems were highly diverse, being based on local system designs, but most focused on respiratory syndromes (figure 2). As respiration is a primary focus of EMS staff in training and triaging, most illnesses reported in EMS data are respiratory. ILI is very common in these data because of the high incidence of acute febrile respiratory infections and because ILI is considered a prodrome for many diseases associated with bioterrorism.^31^ We found only little use of CCD&A-based syndromic surveillance for specific syndromes other than respiratory. Four systems focused exclusively on gastrointestinal syndromes, which mostly reflected food- and water-borne infections [10] [Reference 64 in Supplementary file S1], and diverse disease syndromes were the focus of systems aimed at situational awareness at mass gatherings.

In contrast to the use of CCD&A data for infectious disease surveillance, the literature is extensive on CCD&A-based syndromic surveillance for purposes such as monitoring the health effects of extreme weather events.^32^^,^^33^ In addition, many existing syndromic surveillance systems approximate CCD&A-based syndromic surveillance, e.g. nurse telephone helplines (e.g. British NHS 111) and systems using data from primary care-linked healthcare emergency services (e.g. French SOS Mèdecins, Dutch ICARES);^34–36^ [Reference 74 in Supplementary file S1]. While acknowledging their importance, we were restricted to CCD&A data and therefore did not include such initiatives.

Some main advantages of CCD&A-based syndromic surveillance of infectious diseases emerged from our literature review and interviews. Timeliness was considered a major benefit, though it was not addressed in all publications. Reported time gains varied between a day and two months as compared with hospital data, laboratory data and sentinel primary care data. In addition, a study on prediction of ambulance demand (outside the scope of our literature review) also suggested that infectious diseases may cause seasonal increased demand.^37^ Another showed that regional incidence of influenza and gastroenteritis were predictive of emergency calls in the following week.^38^ However, two aspects need to be considered when evaluating timeliness. First, the timeliness of CCD&A-based surveillance is context dependent, being influenced by the quality and timeliness of the local conventional surveillance systems used as reference data. Second, timeliness is dependent on whether observed trends in CCD&A data can actually be attributed to the disease of interest. Our review found reports of non-detection and delays; in one study, multiple small outbreaks remained undetected by CCD&A data, whereas another study reported a 2-day delay of ambulance data compared to reference data,^10^ [Reference 51 in Supplementary file S1].

Our assessment of validity aspects indicated that the perceived and calculated sensitivity of the CCD&A data in the various publications was sufficient for infectious disease surveillance, as the patterns in CCD&A data corresponded largely with the reference data.^21^^,^^22^^,^^27^ However, multiple publications concluded that sensitivity should be balanced against the PPV to prevent false alerts.^20–23^ Further, since CCD&A data cover a subpopulation with potentially severe illness seeking acute medical care, CCD&A-based syndromic surveillance might complement conventional surveillance systems based on primary care data which usually cover people with milder disease. However, this finding is context dependent. Healthcare systems with higher access hurdles to primary care (low availability, out-of-pocket payments) or weak gate-keeping function tend to see more patients using emergency services for minor ailments. Finally, the grey literature described CCD&A-based syndromic surveillance in Uganda, Haiti and India [References 65–67 in Supplementary file S1], where its use of pre-existing data sources makes it particularly valuable, as low-income countries can lack the public health resources and laboratory tools needed for health surveillance.^39^^,^^40^.

An important drawback of CCD&A-based syndromic surveillance, identified in both publications and interviews, is that it may not detect outbreaks in which few cases are scattered over a large area and long time period, due to background noise and high syndrome baseline rates.^1^^,^^10^^,^^19^^,^^20^ Of course, healthcare data with more specific diagnoses can also face the same problem of missing a small outbreak, especially when a disease is not recognized initially because of non-available or delayed definitive test results. CCD&A-based syndromic surveillance combining spatial and temporal cluster detection has been recommended to detect smaller outbreaks.^10^^,^^22^ Another drawback is that CCD&A data could over-represent susceptible subgroups (e.g. elderly) that are infected more easily and potentially develop more severe disease. Although, the data may indicate the disease burden among specific groups, it probably does not reflect the prevalence of disease in the general population (i.e. the number of uncomplicated cases). A disadvantage of CC-dispatch data, mentioned by some respondents, was its dependence on laypersons’ impressions of emergencies. Ambulance data were considered more accurate due to on-scene professionals but lacking the standardization usually seen in CC-dispatch data. Hence, dispatch data and ambulance data have flaws but seem complementary and most useful when combined. Moreover, structured and algorithm-based call-taking procedures like AMPDS or the Criteria-Based Dispatch (CBD) systems (Nordic Index, Danish Index, Nederlandse Triage Standard, etc.) are designed and continuously validated to translate emergency reporting by laypeople into reliable dispatch information. The accuracy and relevance of any surveillance depends on the standardized and clinically validated call-taking procedures in place at emergency dispatch centres.

Despite its optimistic findings as to CCD&A-based syndromic surveillance, the present overview has some limitations. First, the search terms and the restriction to English articles may have excluded some relevant publications. Second, grey publications were limited to those found via four large organizations, possibly excluding contributions of less well-known organizations. The reviewed publications did not confirm whether alerts generated by CCD&A data were actually caused by the pathogens used as reference data, thereby hampering CCD&A data quality assessment. Moreover, publication bias may have played a role in the predominantly positive results, especially when commercial interests are involved. Finally, as key informants were mainly from Europe and the USA and reasoned within the context of their own location and surveillance design, their observations might not be applicable to other locations.

## Conclusion

This research project sought to provide an overview of the use of CCD&A data in the syndromic surveillance of infectious diseases and its perceived utility in disease detection. The findings of the scoping review imply that that CCD&A data were used in at least 44 studies/systems, but much of this is not reported in peer-reviewed journals and most concern retrospective research of non-continuous systems or data. Most publications focused on respiratory syndromes, with gastrointestinal syndromes as a distant second. The potential utility of CCD&A-data in surveillance of other infectious disease syndromes was rarely discussed and needs further study.

Overall, CCD&A-based syndromic surveillance was reported to have the potential to detect infectious disease outbreaks in a timely manner, but whether detected outbreaks were actually caused by the reference pathogens was not proven. Timeliness was considered a main advantage of CCD&A-based syndromic surveillance, but its pitfalls led many to advise its use in combination with additional data. Nevertheless, CCD&A-based syndromic surveillance makes use of existing, real-time and sometimes highly standardized data sources containing health information. It thereby might enable near real-time and low-cost surveillance of severe cases of infectious diseases, complementing traditional health surveillance. Future assessment of the sensitivity and specificity of CCD&A data by implementation of CCD&A-based surveillance is needed to further elucidate the potential benefit of CCD&A data for syndromic surveillance of infectious diseases.

## Ethics approval and consent to participate

In the Netherlands, medical-academic research that does not subject participants to medical procedures or interventions does not require ethical approval (according to the Central Committee on Research Involving Human Subjects, http://www.ccmo.nl/en/your-research-does-it-fall-under-the-wmo). Our participants were informed of study procedures and gave written and/or verbal consent through their willingness to participate before setting an interview date. Complete transcripts of the interviews were sent to the participants to ensure correctness of the recorded interview.

## Consent for publication

None declared.

## Availability of data and material

Data sharing is not applicable to this article as no datasets were generated or analyzed. Full transcripts of the interviews with key informants are not publicly available to ensure their privacy, but are available from the corresponding author upon reasonable request.

## Supplementary Material

ckz177_Supplementary_Data
