# Supplementary material for: Utility of emergency call centre, dispatch and ambulance data for syndromic surveillance of infectious diseases: a scoping review
Source: Eur J Public Health. 2019 Oct 12;30(4):639–47. doi: 10.1093/eurpub/ckz177 (PMC7446941; doi:10.1093/eurpub/ckz177)
Supplement: ckz177_Supplementary_Data [file ckz177_supplementary_data.zip › ejph-2019-01-srm-0040-File006.docx]

Supplementary 1: References
References 1-40 are in the body of the manuscript.

1. Cady F. The medical priority dispatch system- a system and product overview: <https://www.emergencydispatch.org/articles/ArticleMPDS%28Cady%29.html> (April 2017)
2. Ayala A, Berisha V, Goodin K et al. Public Health Surveillance Strategies for Mass Gatherings: Super Bowl XLIX and Related Events, Maricopa County, Arizona, 2015. Health Security 2016;14(3):173-84.
3. Brunetti ND, Dellegrottaglie G, De Gennaro L, Gaglione A, Di Biase M. 2014 Failed Influenza Vaccination Winter Campaign: Impact on Emergency Medical Service Calls Assessed by Telemedicine. Epidemiology 2015;26(5):e61-e2.
4. Brzezińska-Pawłowska OE, Rydzewska AD, Łuczyńska M, Majkowska-Wojciechowska B, Kowalski ML, Makowska JS. Environmental factors affecting seasonality of ambulance emergency service visits for exacerbations of asthma and COPD. J Asthma 2016;53(2):139-45.
5. Cretikos M, Muscatello D, Patterson J et al. Progression and impact of the first winter wave of the 2009 pandemic H1N1 influenza in New South Wales, Australia. Euro Surveill 2009;14(42).
6. Fishbein D, Sandoval M, Wright C et al. Public Health Surveillance using Emergency Medical Service logs-US-Mexico land border, El Paso, Texas, 2009. MMWR 2010;59(21):649-53.
7. Foldy SL, Biedrzycki PA, Baker BK et al. The public health dashboard: a surveillance model for bioterrorism preparedness. J Public Health Manag Pract 2004;10(3):234-40.
8. Franke F, Coulon L, Renaudat C, Euillot B, Kessalis N, Malfait P. Epidemiologic surveillance system implemented in the Hautes-Alpes District, France, during the Winter Olympic Games, Torino 2006. Euro Surveill 2006;11(12):pii=671.
9. Ohkusa Y, Yamaguchi R, Sugiura H et al. 2008 G8 Hokkaido Toyako Summit Meeting Syndrome Surveillance. Kansenshogaku Zasshi 2009;83(3):236-44.
10. Polkinghorne BG, Muscatello DJ, MacIntyre CR, Lawrence GL, Middleton PM, Torvaldsen S. Relationship between the population incidence of febrile convulsions in young children in Sydney, Australia and seasonal epidemics of influenza and respiratory syncytial virus, 2003-2010: a time series analysis. BMC Inf Dis 2011; 11(1):291.
11. Rosenkötter N, Ziemann A, Riesgo LG-C et al. Validity and timeliness of syndromic influenza surveillance during the autumn/winter wave of A (H1N1) influenza 2009: results of emergency medical dispatch, ambulance and emergency department data from three European regions. BMC Public Health 2013;13(1):1.
12. USA Today. High- tech tool warns of flu outbreaks: <https://www.firstwatch.net/high-tech-tool-warns-of-flu-outbreaks/> (6 July 2016).
13. Barishansky R, O'Connor K. Best practices in emergency medical services. Clinical response planning--syndromic surveillance. Emerg Med Serv 2005;34(3):84.
14. FirstWatch. FirstWatch Trigger Examples & Ideas: https://www.firstwatch.net/wp-content/themes/firstwatch/files/EMS%20and%20Fire/FirstWatch%20Dashboards%20&%20Trigger%20examples_20090113.pdf (6 July 2016)
15. Scott G. Pandemic Flu And 9-1-1. Emergency Number Professional Magazine 2008:19-21.
16. Swift SL, High-Tech Response Keep EMS on Top of Swine Flu Outbreak. Best Practices in Emergency Services 2009;12(7).
17. FirstWatch Media Highlights. 2016a: https://www.firstwatch.net/?s=firstwatch+media+highlights. (6 July 2016).
18. FirstWatch. Early Warning of Flu Epidemic by Real-Time Monitoring of 9-1-1 Call Data, Richmond (Virginia), Oklahoma City and Tulsa (Oklahoma). 2016b: https://www.firstwatch.net/wp-content/themes/firstwatch/files/CS-FluWarning_RichmondVA.pdf. (6 July 2016).
19. Stout T. Creating an EMS Safety Net. Todd Stout builds intricate network via FirstWatch program. In: Kincaid C, editor. 2013.
20. Eyewitness News. Public Safety teams in New Orleans use FirstWatch system for Super Bowl. 2013.
21. Goodwin J. Being Super ready. How technology keeps New Orleans alert, in real time. 2013a. JEMS 2013;38(5):54.
22. FirstWatch. Improving Public Safety and Situational Awareness at Super Bowl XLVII and 2013 Mardi Gras. (2016. https://www.firstwatch.net/wp-content/uploads/2012/02/Special-Events-Case-Study2-2013_FINAL-2.pdf. (6 July 2016).
23. Goodwin J. High Anxiety. National Security Events Take Collaboration, Planning. 2013b. The Journal. 2013:7-8.
24. Cairns C, Potenziani D, Hoit M, Jenkins C, Edgemon S. Novel approach to statewide biosurveillance using emergency medical services (EMS) information. Emerg Health Threats J 2011;4.
25. Jena B, Prasad M, Murthy S, Ramanarao G. Demand pattern of medical emergency services for infectious diseases in Andhra Pradesh—a geo-spatial temporal analysis of fever cases. Indian Emerg J 2010;1(5): 5-8.
26. Jena B. Emergency data based syndromic surveillance in India (SEED) – A GVK EMRI-GEOMED collaborative project [Webinar]: International Society for Disease Surveillance; 2012.
27. Taylor-McCabe K. Contextualizing Data Streams for Infectious Disease Surveillance [Webinar]: International Society for Disease Surveillance; 2013.
28. Stout T, Garza A. Emergency Medical Services (EMS) & Ebola [Webinar]: International Society for Disease Surveillance; 2015.
29. Garza A. From Ebola to Heroin; the Use of EMS Data for Near Real Time Alerting and Surviellance. International Society for Disease Surveillance. 2015. https://www.syndromic.org/storage/documents/isds-conference/2015_Conference/Abstracts/2015_ISDS_Abstract_Book-Part_1.pdf. Accessed 7 July 2016.
30. D’Ortenzio E, Do C, Renault P, Weber F, Filleul L. Enhanced influenza surveillance on Réunion Island (southern hemisphere) in the context of the emergence of influenza A (H1N1) v. Eur Surveill 2009;14(23): pii=19239.
31. Vilain PBA, Dit Cassou J, Morbidelli P, Jacques-Antoine Y, Ristor B,Filleul L. Integrated approach of nonspecific surveillance in Réunion Island. International Society for Disease Surveillance 10th Annual Conference; Atlanta. 2011.
32. Ziemann A, Krafft T, Sala Soler M, Sypniewska P. Country visits. Triple S-AGE project; 2013.
33. Conti S, Kanieff M, Rago G. Triple-S: Inventory of Syndromic Surveillance Systems in Europe. 2012. http://syndromicsurveillance eu/triple-s_inventory_report pdf. (7 July 2016)
34. Santé Publique France. Réseau SOS Médecins / InVS. Santé Publique France. 2015. [http://invs.santepubliquefrance.fr/Dossiers-thematiques/Veille-et-alerte/Surveillance-syndromique-SurSaUD-R/Reseau-SOS-Medecins-InVS (22 Sep 2016](http://invs.santepubliquefrance.fr/Dossiers-thematiques/Veille-et-alerte/Surveillance-syndromique-SurSaUD-R/Reseau-SOS-Medecins-InVS%20(22%20Sep%202016)).
