# Supplementary material for: Utility of emergency call centre, dispatch and ambulance data for syndromic surveillance of infectious diseases: a scoping review
Source: Eur J Public Health. 2019 Oct 12;30(4):639–47. doi: 10.1093/eurpub/ckz177 (PMC7446941; doi:10.1093/eurpub/ckz177)
Supplement: ckz177_Supplementary_Data [file ckz177_supplementary_data.zip › ejph-2019-01-srm-0040-File007.docx]

# Supplementary 2. Search strategy of peer-reviewed and grey literature review

## 1. Search strategy

### Peer-reviewed literature search

Search terms were based on an explorative literature search. The search was performed in Pubmed and was restricted to English language articles published between 1990 and June 2018. Three groups of search terms were established, containing terms related to either Emergency Medical Service (EMS), infectious diseases or public health surveillance in the title and/or abstract (Table A).

**Table A**. Complete list of search terms sorted by the corresponding subgroup.

| **EMS** | **Infectious diseases** | **Public health surveillance** |
| --- | --- | --- |
| Ambulance* | Bacteria* | Early warning |
| Call center*/centre* | Bacterium | Monitor* |
| Call out* | Flu | Surveillance |
| Clinical control center*/centre* | Gastroenteritis | Detection |
| Dispatch* | Gastrointestinal |  |
| Emergency call* | Infectio* |  |
| Emergency service* | Influenza |  |
| Emergency medical service* | Neurologic* |  |
| EMS | Outbreak* |  |
| Telephon* triage | Pathogen* |  |
| 911 call* | Viral |  |
|  | Virologic* |  |
|  | Virus* |  |

The asterisk (*) was used as a wildcard for a selection of the search terms to allow end-truncation.

In the initial search, the search terms within the subgroups were linked using Boolean operator “OR”, and the subgroups as a whole were combined with Boolean operator “AND”, leading to results that contained at least one of the search terms in each subgroup. To prevent overlooking of relevant articles missing the surveillance related search terms (third column) a second search was done combining only the ‘EMS’-column and the ‘infectious diseases’-column. The search terms ‘neurologic(al)’ and ‘telephone/telephonic triage’ were excluded from this second search, due to their lack of specificity resulting large numbers of hits. However, not to disregard these terms. In a third search ‘Neurologic(al)’ was combined with one or more search terms in the ‘EMS’-column and the ‘infectious diseases’-column; the same was done for ‘telephone/telephonic triage’.

**Table B**. Inclusion and exclusion criteria of the search strategy

| **Inclusion criteria** | **Exclusion criteria** |
| --- | --- |
| English language | Published before 1990 |
| All study designs | Research focused on nurse help lines, NHS, telemedicine |
| Research focused on surveillance | Research focused on emergency department data |
| Descriptions and reports of CCD&A-based syndromic surveillance systems and studies | Reviews or general descriptions about CCD&A-based syndromic surveillance |
|  | Research focused on non-infectious diseases or medical conditions |

The inclusion criteria in the scoping review related to the use of CC-dispatch- and/or ambulance (CCD&A) data in the context of at least one infectious disease (outbreak) or disease outcome. Publications which did not mention the use of CCD&A-data in the context of surveillance were excluded. Potential relevant and doubtful articles were read completely by three researchers (SD, JD, LA) to decide whether or not the publication was suitable for inclusion in the scoping review.

## Grey literature search

Because of the large number of hits in the initial search on the Internet, the search was restricted to publications from FirstWatch, the International Society for Disease Surveillance, Triple-S and SIDARTHa. Only publications which met the above search terms, were written in English language and were published between 1990 and June 2018 were selected. A broad range of publication types were explored, including news items, webinars, conference abstracts, and project reports. The SIDARTHa publications did not result in additional studies or system descriptions.

2. Selecting articles
All search results were screened by title and abstract by two reviewers (JD, SD/LA). Subsequently, full-text assessment of the potential relevant and doubtful publications was conducted by three researchers (JD, SD, LA). As is common for scoping reviews, inclusion and exclusion criteria were refined as familiarity with the available literature increased during the search process (Table B).

## 3. Charting information and collating, summarizing and reporting results

From the relevant literature we reported study characteristics and we used these to gain an impression of the timeliness and validity of the published CCD&A-data. Data-charting was performed on the selected publications and documents: we extracted data of interest comprising disease or event for surveillance, data type (CC-dispatch, ambulance) used, data coverage, coding system used, symptom codes, detection methods, data sources, timing of data capturing, generation of alerts, reference data used, and outcome. In addition, we charted descriptive information including year of publication, location of the system or study, study period, and study design. Publications describing a surveillance system or study in detail, addressing most of the topics of interest were considered as studies of primary interest. Publications providing a more general explanation of CCD&A-based syndromic surveillance were used as background information.
