# Supplementary material for: Utility of emergency call centre, dispatch and ambulance data for syndromic surveillance of infectious diseases: a scoping review
Source: Eur J Public Health. 2019 Oct 12;30(4):639–47. doi: 10.1093/eurpub/ckz177 (PMC7446941; doi:10.1093/eurpub/ckz177)
Supplement: ckz177_Supplementary_Data [file ckz177_supplementary_data.zip › ejph-2019-01-srm-0040-File008.docx]

**Supplementary table S1**. Study characteristics of the selected peer-reviewed publications

| **First author, year** | **Country** | **Disease /event**  **(syndrome)** | **Data type** | **Study period** | **Data source** | **Data coverage** | **Data capturing** | **Reference data** | **Data coding** | **Detection methods** | **Generation of alerts** | **Outcome** |
| --- | --- | --- | --- | --- | --- | --- | --- | --- | --- | --- | --- | --- |
| Ayala, 2016 [Sup1-ref42] | USA | Public health emergencies (gastrointestinal and respiratory syndromes) | CC-dispatch data & Ambulance data | 22 Jan –  6 Feb 2015 | Phoenix and Scottsdale fire and EMS services. Arizona Prehospital Information and EMS Registry System (AZ-PIERS) | Maricopa County, Arizona | Daily | n/a | Unspecified | Unspecified | Unspecified | Data from emergency dispatching center to limited. No health concerns from AZ-PIERS reports |
| Bork, 2006 [19] | Denmark | Influenza  (ambulance transport frequency) | CC-dispatch data | Jan 2000 –  Aug 2005 | CC-dispatch data from six regions in Denmark | 5.4 million people | Daily | National Influenza Sentinel Registration | n/a | Space dynamic model with Kalman smoother | Exceeding upper limit of prediction intervals at 95% and 99% | a) Influenza outbreak of 2003 detected in timely fashion  (1 alert prior to outbreak, 13 alerts during outbreak).  b) All outbreak scenarios detected. |
| Brunetti, 2015 [Sup1-ref43] | Italy | Influenza-related CVD† (n/a ‡) | Ambulance data | Dec 2014 –  Jan 2015 | Unspecified | Unspecified | Daily | CC-dispatch data and ambulance data from  Dec 2013 –  Jan 2014 | Unspecified | n/a | n/a | a) Increased number of CVD responses in 2014-2015  (December 2014: + 21%,  January 2015: + 27%).  b) Overall increase in EMS-calls  (January 2015: + 7%)  c) Increased number of CVD responses at 2014 influenza peak (+25-29%) |
| Brzezińska-Pawłowska, 2016 [Sup1-ref 44] | Poland | Exacerbations of asthma & COPD | Ambulance | Jan 2007 -  Dec 2008 | Ambulance Emergency Service (AES) Database Lodz | Lodz, ~750,000 | n/a | National Institute of Public Health reported number of influenza or ILI infections | Unspecified | Spearman’s correlation test | n/a | Number of influenza cases was parallel to number of asthma/COPD AES visits. |
| Coory, 2009 [20] | Australia | ILI  (respiratory problems, breathing problems) | CC-dispatch data | Jan 1997 –  Dec 2005 | CC-dispatch data from Melbourne | 3.8 million people | Weekly | GP^○^ sentinel data, locum service data | AMPDS^♠^ | CUSUM ^¤^ | h/2 with h = 10 | a) CC-dispatch data reflected ILI trends (including peak intensity).  b) Influenza detected between 1 week and 2 months earlier than or simultaneous to GP sentinel data in peak years 1997, 1998 and 2003 (possibility for false positives). |
| Cretikos, 2009 [Sup1-ref45] | Australia | Influenza (ambulance activity) | CC-dispatch data | 2009  (timespan unspecified) | CC-dispatch data from Sydney regions | 4,4 million people | Minutely | Sydney CC-dispatch data from 2008 | Unspecified | n/a | n/a | a) Rise in ambulance activity above seasonally expected levels. Peak occurred one week prior to hospital and laboratory data of confirmed  A(H1N1)v cases.  b) Overall dispatch call frequency: +13%  Calls for breathing  problems: + 56%  b) Other increased call types: headache, person ill, unconscious/fainting, fitting/ convulsion, chest pain. |
| Fishbein, 2010 [Sup1-ref46] | USA | Notifiable infectious diseases  (unspecified) | CC-dispatch data & ambulance data | Jan 2009 –  Dec 2009 | EMS dispatch- and response logs from 4 ports of entry in El Paso | 31.5 million crossings/ year | Daily | CDC’s Quarantine and Activity Reporting System (QARS) | Unspecified | Commercial software (FirstWatch) | Any case with symptoms potentially indicative of an infectious disease | a) Use of EMS dispatch and response logs doubled  reports of infectious diseases. b) Number of identified reportable diseases increased by more than five-fold. |
| Foldy, 2004 [Sup1-ref47] | USA | Several high-profile entertainment events including the Major League All Star Game  (unspecified) | CC-dispatch data | June 25, 2002 – July 22, 2002 | Unspecified | Unspecified | Daily | n/a | Unspecified | Unspecified | Increase in cases of two standard deviations above baseline | No outbreaks detected |
| Franke, 2006 [Sup1-ref48] | France | 2006 Olympic Winter Games  (unspecified) | CC-dispatch data | January 30, 2006– May 15, 2006 | Haute-Alpes Emergency Ambulance Service (SAMU^¥^) | Unspecified | Daily | SAMU CC-dispatch data from 2005 | Unspecified | Unspecified | Unspecified | a) No adverse health events identified  b) Activities of SAMU observed similar to previous year |
| Greenko, 2003 [15] | USA | ILI  (respiratory distress, difficulty breathing sick adult, sick pediatric) | CC-dispatch data. | Jan 19. 1999  (one day) | Emergency department medical charts  (besides symptoms, these charts were reviewed for information on ambulance utilization and EMS call type) | All emergency department visits in six high volume New York City hospitals | n/a | n/a | n/a | n/a | n/a | a) Biases in ambulance data:  older population, more severe disease expressions.  b) Predictive value positive of ILI call types: 22% |
| Haas, 2011 [21];  EPA,2014 [25]** | USA | Water contamination clusters  (abdominal pain, allergies, breathing problems, cardiac- or respiratory distress, headache, hemorrhaging, fainting, possible stroke, seizures, unconsciousness, burns and blisters) | CC-dispatch data & ambulance data | Jan 2008 –  Jun 2010 | CC-dispatch data from Cincinnati Fire Department (CFD), ambulance data from CFD paramedics and Emergency Medical Technicians. | Cincinnati city. 760.000 | Hourly | Other public health data sources in the surveillance system. | AMPDS. | Space-time permutation scan statistics (dispatch), EARS^¥^, CUSUM (ambulance data) | Significant cluster (n>16,  α = 0.0250) | All alerts were false. |
| Mostashari,  2003 [22] | USA | ILI*  (respiratory distress, difficulty breathing, sick, sick pediatric) | CC-dispatch data | Sep 1995 –  Apr 1998  Sep 1999 –  Jan 2003 | New York City Fire Department EMS^●^ | Unspecified | Daily | Weekly virus isolation data of WHO collaborating labs in New York City | Unspecified | Linear regression of the EMS ILI call rate | Exceeding upper limit of prediction intervals at 95% and 99% | Prospective influenza detection 2-3 weeks earlier than traditional surveillance |
| Ohkusa, 2011 [Sup1-ref49] | Japan | Infectious disease outbreaks | CC-dispatch data | 23 June –  23 July 2008 | Seven Fire Departments in Toyako area, Hokkaido | Hokkaido, Toyako | Hourly | Disease surveillance report data of National Institute of Infectious Diseases. | Bestoru | Poisson regression on number of ambulance transfers | Aberration from expected number: low level <2,5%; medium level 1%; high level 0.1%. | No suspected outbreaks observed during G8, 7 high aberrations investigated based on aberrance in ambulance utilization. |
| Polkinghorne, 2011 [Sup1-ref50] | Australia | Febrile convulsions  (fitting or convulsions in children under 6) | CC-dispatch data | Jul 1, 2006 –  Apr 30, 2010 | Ambulance Service of New South Wales’ computer-  aided CC-dispatch database for Sydney | 4.58 million people | Daily, weekly | New South Wales emergency department data collection on the Health Outcomes  and Information Statistical Toolkit (HOIST) database for 35 public hospitals in Sydney | Unspecified | n/a | n/a | 1) Significant association between population incident rate of emergency department presentations of ILI and population incident rate of emergency calls for fittings and convulsions.  2) Associations strongest if convulsions preceded ILI presentations with one week |
| Rosenkötter, 2013 [Sup1-ref51], Rosenkötter, 2010 [26]**, | Austria | ILI  (breathing problems, sick person, acute nasopharyngitis/  pharyngitis/  laryngitis/upper respiratory infection, influenza, pneumonia, cough, fever) | CC-dispatch data & ambulance data | Jan- Dec 2009 | Dispatch center Tirol | Dispatch: 381.456 people. Ambulance: 99.394 people. | Daily | Sick leaves due to acute respiratory illness, obtained from Tirolean health insurance | CC-dispatch data: AMPDS^‡^  Ambulance data:  ICD-10^●^ | CUSUM-P SaTScan | CUSUM-P:  Exceeding threshold *h,* determined by Lucas table.  SaTScan:  P-value of cluster < 0.05 | CC-dispatch data: time gain: 1-5 days. Ambulance data: time gain: 25 days. |
| Ziemann, 2014 [10] |  | Gastrointestinal outbreaks |  | Jan 2007- Dec 2009 |  |  |  | Notifiable surveillance reports of foodborne diseases in Austria |  | CUSUM-N;  CUSUM-P | Threshold value retrieved from Lucas table | One confirmed norovirus outbreak detected in Tirolean ambulance data. Two norovirus outbreaks remained unidentified. |
| Rosenkötter, 2013 [Sup1-ref51], Rosenkötter, 2010 [26]** | Belgium | ILI | Ambulance data | Jan-Dec 2009 | Ministry of Health, Belgium | 10,500,000 people. | Daily | Notified seasonal and H1N1 influenza, obtained from Belgium National Influenza Center | ICD-9 ^♣^, | CUSUM-P, SaTScan | CUSUM-P:  Exceeding threshold *h,* determined by Lucas table.  SaTScan:  P-value of cluster < 0.05 | CC-dispatch data peaked 11 days ahead of reference data. Belgian ambulance data was two days late. |
| Ziemann, 2014 [10] |  | Gastrointestinal outbreaks |  | Jan 2007- Dec 2009 |  |  |  | Notifiable surveillance reports of foodborne diseases in Austria |  | CUSUM-N  CUSUM-P | Threshold value retrieved from Lucas table | No outbreaks detected. |
| Rosenkötter, 2010 [26]** | Germany | ILI | Ambulance data | Jan-Dec 2009 | Emergency physician prehospital emergency, district Göppingen | 255.807 people | Daily | Notified influenza A cases, obtained from Robert Koch Institute | ICD-10, MIND-2^$^ | CUSUM-P, SaTScan | CUSUM-P:  Exceeding threshold *h,* determined by Lucas table.  SaTScan:  P-value of cluster < 0.05 | Time gain: unspecified |
| Ziemann, 2014 [10]; Rosenkötter, 2010 [26]** |  | Gastrointestinal outbreaks |  | Jan 2007 –  Dec 2008 | Emergency physician ambulance service, Göppingen |  |  | Notifiable surveillance reports of foodborne diseases in Austria, Belgium and Germany |  | CUSUM-N, CUSUM-P | Threshold value retrieved from Lucas table | No outbreaks detected. |
| Schull, 2004 [12] | Canada | Influenza and respiratory illnesses | Ambulance diversion data | Jan 1996 –  April 1999 | Ambulance diversion conditions from 18 Eds | 2.3 million people | Weekly | Laboratory confirmed respiratory infections, Canada’s Laboratory for Disease Control. ED visits, Ontario Health Insurance Program. | n/a | n/a | n/a | Influenza seasons are associated with increased ED ambulance diversion. |
| Shimantani. 2015 [23] | Japan | 2013 Sports Festival, Tokyo  (chief complaints: fever, diarrhea, vomiting, rash) | Ambulance data | September 21, 2013- October 28, 2013 | Tokyo Metropolitan Institute of Public Health | Tokyo metropolitan area (600.000 ambulance transfers per year) | Daily | n/a | Unspecified | Unspecified | Significant statistical increase in cases based on historical data | 38 high level aberrations for hematemesis/epistaxis (n=30), nausea/vomiting (n=4), dizziness (n=3) and unconsciousness (n=1). None required follow-up. |
| Todkill [24] | England |  | CC-dispatch data | Sept 2015 – March 2016 | West Midlands Ambulance Service | 5.6 million people | Daily | CC-dispatch data Aug 2013 – Aug 2015, national respiratory syncytial virus and influenza virus laboratory surveillance | Unspecified | Rising Activity Multi Level Mixed Effects, Indicator Emphasis (RAMMIE) model | ‘Spike alarms’ and ‘historic alarms, compared to recent and baseline call numbers | 49 statistical alarms. Timely detection of seasonal changes in patterns of respiratory calls and increased calls coinciding with seasonal events |
| Tsubokura,  2010 [25] | Japan | Febrile illness | Ambulance data | 16 April – 16 June 2009 | Fire Prevention Bureau of Kobe | Kobe | Daily | *Preepidemic stage* data | Unspecified | n/a | n/a | H1N1 influenza pandemic did nog have a great impact on (supply and demand of) EMS system |

CC-dispatch= call center dispatch; ILI = Influenza-like illness; ○AMPDS = Advanced Medical Priority Dispatch System; ¥ EARS=Early Aberration Response System; † CVD = Cardiovascular Disease; ♠ ICD-9 = International Classification of Diseases, 9^th^ revision; ‡ n/a = not applicable; ♣ ICD-10 = International Classification of Diseases, 10^th^ revision; ● EMS = Emergency Medical Services; ♦MIND-II = Minimaler Notartzdatensatz (minimum data set for emergency physicians); ○ GP = General Practitioner, i.e. primary care; $ CUSUM = Cumulative Sum (Statistical test). **Publication found in grey literature adding information to peer-reviewed publication.
