# Supplementary material for: Utility of emergency call centre, dispatch and ambulance data for syndromic surveillance of infectious diseases: a scoping review
Source: Eur J Public Health. 2019 Oct 12;30(4):639–47. doi: 10.1093/eurpub/ckz177 (PMC7446941; doi:10.1093/eurpub/ckz177)
Supplement: ckz177_Supplementary_Data [file ckz177_supplementary_data.zip › ejph-2019-01-srm-0040-File009.docx]

**Supplementary table S2**. Characteristics of CCD&A-based syndromic surveillance activities of FirstWatch in grey literature

| **First author/**  **organization, year** | **Country** | **Disease/ Event (syndrome)** | **Data type** | **System activity period** | **Data source** | **Data coverage** | **Data capturing** | **Data coding** | **Detection methods** | **Generation of alerts** | **Outcome** |
| --- | --- | --- | --- | --- | --- | --- | --- | --- | --- | --- | --- |
| USA Today, 2003 [Sup1-ref52] | USA | Influenza  (fever > 100 degrees, chills, headaches, sore throat, stuffy nose, cough, extreme tiredness, body aches) | CC-dispatch data | Unsp | Unsp | Unsp | Unsp | Unsp | Unsp | Unsp | Alerts in  Oklahoma: Nov 20  Tulsa: Nov 22 |
| Barishansky, 2005 [Sup1-ref53] | USA | Influenza (unspecified) | CC-dispatch data | Unsp | Unsp | 14 million people | Unsp | Unsp | Unsp | Unusual increases in volume or clustering patterns | Identification of influenza outbreaks in Texas, Virginia and Oklahoma |
| FirstWatch, 2007 [Sup1-ref54] | USA | Influenza  (respiratory problems, abdominal pain, headache (and more, unspecified),  food poisoning  (unspecified) | CC-dispatch data | Unsp | Unsp | Unspecified | Unsp | Unsp | Influenza:  Unspecified  Food poisoning: CUSUM | Unsp | Influenza: n/a food poisoning: CUSUM alert 3 hours after event |
| Scott, 2008 [Sup1-ref55] | USA | Influenza  (unspecified) | CC-dispatch data | Unsp | Unsp | Unsp | Unsp | AMPDS† | Unsp | Unusual trends and patterns indicative of outbreaks | n/a |
| Simon, 2009 [Sup1-ref56] | Canada | Pandemic influenza  (i.e. fever, febrile, Mexico) | CC-dispatch data | Unsp | Toronto EMS | Unsp | Unsp | Unsp | Unsp | Exceeding FirstWatch threshold level | Two calls identified directly after adding term ‘Mexico’ |
| FirstWatch, 2016a [Sup1-ref57] | USA | Influenza and bioterrorism attacks  (breathing problems, chest pain or other symptoms suggestive of flu and bioterrorism) | CC-dispatch data | Unsp | Oklahoma: EMS Authority  San Diego: Kerney Mesa Dispatch Center Pinellas County: unspecified | Unsp | Real-time | Unsp | Unsp | Unsp | n/a |
| FirstWatch, 2016b [Sup1-ref58] | USA | Influenza  (Oklahoma City: Unspecified Tulsa: Unspecified  Richmond: Abdominal pain, breathing problems, sick person) | CC-dispatch data | Unsp | Oklahoma: Oklahoma EMS Authority  Richmond: Richmond Ambulance Authority, | Oklahoma & Tulsa:  110.000 annual calls  Richmond:  50.000 annual calls | Unsp | Unsp | Unsp | Unusual increases in volume or clustering patterns | Alerts in  Oklahoma City: Nov 16, 2003  Tulsa: Nov 20, 2003  Richmond:  Unspecified |
| Stout, 2011 [Sup1-ref59] | USA | Conference attended by Barack Obama  (unspecified) | CC-dispatch data | Unsp | Unspecified | Unsp | Unsp | Unsp | Unsp | Unsp | Unsp |
| Eyewitness News, 2013; Goodwin, 2013a; FirstWatch, 2016 [Sup1-ref60-62] | USA | Mardi Grass and Super Bowl XLVII  (i.e. seizure, abdominal pain, breathing problems, sudden headaches, fainting episodes) | CC-dispatch data | Unsp | New Orleans Dispatch Center | All police, fire and EMS-events city wide and in hospitality zone, hotel zone and convention center zone | Real-time | Unsp | Unsp | Exceeding FirstWatch threshold level | No alerts |
| Goodwin, 2013b [Sup1-ref63] | USA | Republican and Democratic National Convention  (fever, rash, cough, altered mental status, bloody diarrhea, seizure) | CC-dispatch data | Aug-Sep 2012 | Tampa: Pinellas County Central Dispatch  Charlotte: Mecklenburg Emergency Medical Service Agency | Unsp | Real-time | AMPDS, ePCR | Unsp | Unsp | No alerts |

CCD&A= Call Center Dispatch & Ambulance; CC-dispatch= call center dispatch; n/a = not applicable; Unsp = unspecified; EMS= Emergency Medical Services; AMPDS = Advanced Medical Priority Dispatch System; ePCR = electronic Patient Care Record; CUSUM = Cumulative Sum (statistical test);
