# Supplementary material for: Utility of emergency call centre, dispatch and ambulance data for syndromic surveillance of infectious diseases: a scoping review
Source: Eur J Public Health. 2019 Oct 12;30(4):639–47. doi: 10.1093/eurpub/ckz177 (PMC7446941; doi:10.1093/eurpub/ckz177)
Supplement: ckz177_Supplementary_Data [file ckz177_supplementary_data.zip › ejph-2019-01-srm-0040-File010.docx]

**Supplementary table S3**. Characteristics of CCD&A-based syndromic surveillance activities in ISDS conference publications in grey literature

| **Organization, year** | **Country** | **Disease/ Event**  **(syndrome)** | **Data Type** | **System activity period/ study period** | **Data source** | **Data coverage** | **Data capturing** | **Reference data** | **Data coding** | **Detection methods** | **Generation of alerts** | **Outcome** |
| --- | --- | --- | --- | --- | --- | --- | --- | --- | --- | --- | --- | --- |
| Cairns, 2011 [Sup1-ref64] | USA | Gastrointestinal illness (unspecified) | CC-dispatch data | 2009-2010 | PreMIS | North Carolina | Unsp | Unsp | Unsp | CUSUM and text proportion analysis (TAP) | Exceeding baseline mean by 3 standard deviations | Time gain CUSUM:  7 days  Time gain TAP:  56 days  (reference data unspecified) |
| Jena, 2010 [Sup1-ref65] | India | Fever | CC-dispatch data | Apr 2007 -  March 2009 | GVK-EMRI | Three districts in State Andhra Pradesh | n/a | Estimated prevalence rate of fever (survey) | Unsp | n/a | n/a | Seasonal, age and socio-economic variation in demand. |
| Jena, 2012 [Sup1-ref66] | India | Model phase:  Fever  (unspecified) Test phase:  Dengue (unspecified) | CC-dispatch data | Model phase:  Apr 2007 –  Jul 2009  Test phase:  Aug 2009-  Sep 2010 | GVK-EMRI data warehouse | Model phase:  143.116 calls  Test phase:  47.233 calls | 2-3 hourly | Model phase:  n/a  Test phase:  national newspapers | Unsp | Model phase:  Exceeding upper limit at 95% and 99% confidence interval  Test phase:  Exceeding upper limit at 95% and 99% confidence interval,  EARS, SaTScan | Model/Test phase: Exceeding upper limit of 95% and 99% confidence interval  Test phase:  EARS, SaTScan | Time gain: 15 days |
| Taylor-McCabe, 2013 [Sup1-ref67] | Uganda, | Ebola  (unspecified) | Ambulance data | 2011  (period unspecified) | Unsp | Unsp | Unsp | n/a | Unsp | Unsp | Unsp | n/a |
|  | Haiti | Cholera (unspecified) | Ambulance data | Oct 20 –  Dec 3,2010 |  |  |  |  |  |  |  |  |
| Stout, 2015; Garza, 2015 [Sup1-ref68-69] | USA | Ebola (Travel: includes mainly countries in West Africa  Symptoms: free text or pre-set symptoms indicative of ILI, gastrointestinal problems and hemorrhaging, risk exposures) | CC-dispatch data | Oct 2014 –  Mar 2015 | Unsp | > 40 EMS agencies | Unsp | n/a | AMDPS , ePCR, other CAD systems | Unsp | Any case compliant with Ebola triggers | 1532 alerts. All tested negative for Ebola. |

CCD&A= Call Center Dispatch & Ambulance; CC-dispatch= call center dispatch; n/a = not applicable; Unsp = unspecified; PreMIS= Pre-hospital Medical Information System; AMPDS = Advanced Medical Priority Dispatch System; ILI = Influenza-like Illness; GVK-EMRI = Emergency Management and Research Institute; ePCR = electronic Patient Care Record; CUSUM = Cumulative Sum (statistical test); AZ-PIERS = Arizona Pre-hospital Information and EMS Registration System; CAD = Computer Aided Dispatch; EARS= Early Aberration Reporting System;
