# Supplementary material for: Utility of emergency call centre, dispatch and ambulance data for syndromic surveillance of infectious diseases: a scoping review
Source: Eur J Public Health. 2019 Oct 12;30(4):639–47. doi: 10.1093/eurpub/ckz177 (PMC7446941; doi:10.1093/eurpub/ckz177)
Supplement: ckz177_Supplementary_Data [file ckz177_supplementary_data.zip › ejph-2019-01-srm-0040-File011.docx]

**Supplementary table S4**. Characteristics of CCD&A-based syndromic surveillance activities in Triple-S publications in grey literature

| **First author, year** | **Country** | **Disease/ Event (symptom codes)** | **Data Type** | **System activity period** | **Data source** | **Data coverage** | **Data capturing** | **Reference data** | **Data coding** | **Detection methods** | **Generation of alerts** | **Outcome** |
| --- | --- | --- | --- | --- | --- | --- | --- | --- | --- | --- | --- | --- |
| D’Ortenzio, 2009; Vilain, 2011 [Sup1-ref70-71] | Réunion Island | Pandemic influenza (ILI) | CC-dispatch data | Week 19 2009 –  week 1 2010 | Réunion Island EMS^‡^ regulation center | Unspecified | Unspecified | Sentinel GPs | Unsp | Unspecified | Unsp | Peak occurred at same time as sentinel data |
| Ziemann, 2013 [Sup1-ref72][28] | Border region of Belgium, the Netherlands and Germany | n/a (unspecified) | CC-dispatch data | Planned | Unsp | Unspecified | Daily | Unsp | Unsp | Descriptive statistics, CUSUM and spatial analysis | Unsp | n/a |
| Conti,  2012 [Sup1-ref73] | Belgium | n/a (asthma, ILI, neurological symptoms, respiratory illness) | Ambulance data | Planned:  Jan 2015 and Jan 2016 | Unsp | National | Daily | Unsp | ICD-9, ICD-10 | Unspecified | Unsp | n/a |
| Ziemann,  2013; Conti, 2012; [28,29] | Hungary | n/a (asthma, hemorrhagic illness, ILI, cutaneous laesion, lymphadenitis, neurological symptoms, rash, respiratory illness, shortness of breath, specific infection, sun/heat stroke, blood poisoning, botulism-like illness, diarrhea, death, enterovirus-related infection, fever, gastrointestinal illness) | CC-dispatch data | Planned:  Jan 2012 | All 20 dispatch centers | National | Daily | Unsp | ICD-10 | Descriptive statistics, regression analysis, CUSUM and spatial analysis | Unsp | n/a |

CCD&A= Call Center Dispatch & Ambulance; ILI= Influenza-like-Illness; CC-dispatch= call center dispatch; n/a = not applicable; Unsp = unspecified; EMS = Emergency Medical Services; GP= General Practitioner; ICD-9 = International Classification of Diseases, 9^th^ revision; ICD-10 = International Classification of Diseases, 10^th^ revision; CUSUM = Cumulative Sum (statistical test)
