# Supplementary material for: Utility of emergency call centre, dispatch and ambulance data for syndromic surveillance of infectious diseases: a scoping review
Source: Eur J Public Health. 2019 Oct 12;30(4):639–47. doi: 10.1093/eurpub/ckz177 (PMC7446941; doi:10.1093/eurpub/ckz177)
Supplement: ckz177_Supplementary_Data [file ckz177_supplementary_data.zip › ejph-2019-01-srm-0040-File012.docx]

**Supplementary table S5.** Perceived usefulness of CCD&A-based syndromic surveillance

| **Main theme** | **Sub themes** | **Quotes** |
| --- | --- | --- |
| Usefulness: general EMS^*^-based syndromic surveillance | Useful | *“To us, it was a secondary dataset, with the hospital ER [emergency room] visits being primary. It was largely duplicative of that dataset, but there are some emergency medical service visits that don’t make it to the hospital, so those could be useful.” (CCD&A^†^-based surveillance employee 1)* |
|  | Not useful | *“Our ED^‡^-data comes in twice a day and the ambulance data comes in once a day I believe, but it seems like the data is always about two days behind, so.” (CCD&A-based surveillance employee 2)* |
| Usefulness: EMS-based syndromic surveillance for infectious disease detection | Useful | *“Yes, I am convinced. For example, even with aggregated data, we detected and followed the 2009 A(H1N1) influenza outbreak thanks to emergency data.” (Researcher 1)* |
|  | Doubtful use | *“Uhm, well, talking about the dispatch data you always have to keep in mind that it is based on laypersons and on their interpretation of symptoms. So it can only be a collection of unspecific symptoms and that can give you a suspicion of infectious diseases, but not more.” (EMS-healthcare worker 2)* |

CCD&A= Call Center Dispatch & Ambulance; EMS = Emergency Medical Services; ED = Emergency Department.
