# Supplementary material for: Utility of emergency call centre, dispatch and ambulance data for syndromic surveillance of infectious diseases: a scoping review
Source: Eur J Public Health. 2019 Oct 12;30(4):639–47. doi: 10.1093/eurpub/ckz177 (PMC7446941; doi:10.1093/eurpub/ckz177)
Supplement: ckz177_Supplementary_Data [file ckz177_supplementary_data.zip › ejph-2019-01-srm-0040-File013.docx]

**Supplementary table S6.** Perceived advantages and disadvantages of using CCD&A-data in syndromic surveillance

| **Data type** | **Main themes** | **Sub themes** | **Quotes** |
| --- | --- | --- | --- |
| Cc-dispatch data and ambulance data | Attitudes towards cc-dispatch- and ambulance data:  general surveillance | Advantages | *“It’s there. It’s existing, it’s electronic, it’s collected every hour at least or you can have it on a daily basis. It’s, uhm, these are advantages of syndromic data in general that you use data that is already collected and you can just tap on it without having to collect data.” (Researcher 2)* |
|  |  | Disadvantages | *“I think your big problem is not about EMS^*^, your problem would be that, uhm, professionalizing has led to isolation between the different silos. So information is not shared enough and that means that nobody has the whole picture, has the whole process information.” (EMS-healthcare worker 2)* |
|  | Attitudes towards cc-dispatch- and ambulance data:  Infectious disease surveillance | Advantages | *“The only thing is that it buys you time because you might get an earlier warning. And this is for, uhm, especially for infectious disease control and containment, essential. The earlier you get to your start in containment, the larger the chances are that you can really get the outbreak under control.” (Researcher 3)* |
|  |  | Disadvantages | *“Ambulances won’t go to schools if they start coughing in kindergarten, you know. It depends. The outbreak needs to be severe enough for the ambulance to come and anticipate.” (CCD&A-based surveillance employee 4)* |
| Cc-dispatch data | Cc-dispatch data in general surveillance | Advantages | *“In a way I think the data from the dispatch center is less biased [than ambulance data], because you strictly follow a standard protocol and they don’t see the patient*.*” (Researcher 3)* |
|  |  | Disadvantages | *“(…) in EMS systems you are working with very little information from the start. So the moment a call comes in, you’re connected with a non-professional. You’re not getting any measurements and it is with other variables that you need to get information.” (EMS-healthcare worker 2)* |
|  | Cc-dispatch data in infectious disease surveillance | Advantages | *“Practitioners have to report special diseases to the authorities, but this reporting system needs one to two weeks and they find there is a problem. And this EMS-data you have it on the same or on the next day. It’s much faster.”*  *(EMS-healthcare worker 3)* |
|  |  | Disadvantages | *“Over-diagnosis giving inflated figures, especially when using dispatch data. I was sent to a number of potential Ebola cases as a paramedic and some of these differential diagnoses were made with the patient stating 'I think I have Ebola.' This illustrates the public response when there is an epidemic occurring, that they are prone to panic when they get a fever.” (EMS-healthcare worker 4)* |
| Ambulance data | Ambulance data in general surveillance | Advantages | *“And the moment the ambulance crew enters the location of the patient, they are with more equipment. They can even have blood analytic, blood analysis machines with the. They could do a lot more, because they’re medically trained. (…) they can give you more specific information” (EMS-healthcare worker 2)* |
|  |  | Disadvantages | “*Yes, and that in itself is an issue because there’s no universal for ePCR^†^ data and there’s different companies that do it.” (Researcher 4)* |
|  | Ambulance data in infectious disease surveillance | Advantages | “(…*) through the EMS-data you could get a sense of what was happening and where it’s happening and then you can also get a sense of where the patient is transported to, which will help you understand the burden on any individual hospital or couple of hospitals which can help for response purposes if the event is big enough”*  *(EMS-healthcare worker 2)* |
|  |  | Disadvantages | *“EMS-personnel are usually trained to get the patient to the hospital alive and they’re not especially trained in recognizing some kind of infectious diseases.” (EMS-healthcare worker 1)* |

CCD&A= Call Center Dispatch & Ambulance; Cc-dispatch= call center dispatch; EMS= Emergency Medical Services; ePCR= electronic Patient Care Record, a file containing patient information established by ambulance personnel
