# Supplementary material for: Utility of emergency call centre, dispatch and ambulance data for syndromic surveillance of infectious diseases: a scoping review
Source: Eur J Public Health. 2019 Oct 12;30(4):639–47. doi: 10.1093/eurpub/ckz177 (PMC7446941; doi:10.1093/eurpub/ckz177)
Supplement: ckz177_Supplementary_Data [file ckz177_supplementary_data.zip › ejph-2019-01-srm-0040-File014.docx]

**Supplementary table S7**. Alternative uses and suggested improvements for CCD&A-based syndromic surveillance

| **Main themes** | **Sub themes** | **Quotes** |
| --- | --- | --- |
| Alternative uses | Situational awareness | *“Right, you know it’s definitely worth monitoring if we know there’s a large scale event happening. Like last year the MBA All Star game was supposed to be in Charlotte. So, uhm, that attracted a lot of people and a lot of tourists to the city. It would be something good to monitor during the event to see if anything is happening” (CCD&A^*^-based surveillance employee 2)* |
|  | EMS^†^-logistics | *“I think what is also important is that the same mechanism that we are using for syndromic surveillance, is also used for operating the system. Because in a way you define thresholds, you try to understand how your system is responding, and the better you understand the actual workload and also the medical resources behind your workload, the better you can respond.” (Researcher 3)* |
| Improvements | Data integration | *“I never read about a feedback loop looking at the first patient, the second and the third who already have processed further on in the health pathway to get that information back in the system. Because they would have already been in the ambulance, they would have done all the diagnostics, they would have a lot more information” (EMS-healthcare worker 2)* |
|  | EMS-facilities | *“I think it would be a benefit for the responders to have some kind of quick test or quick diagnosis that can be done in the field” (EMS-healthcare worker 1)* |

* CCD&A = Call Center Dispatch and Ambulance; † EMS = Emergency Medical Services.
